# Whole-Genome Sequencing of Mycoplasma mycoides subsp. mycoides Italian Strain 57/13, the Causative Agent of Contagious Bovine Pleuropneumonia

**DOI:** 10.1128/genomeA.00197-15

**Published:** 2015-03-26

**Authors:** M. Orsini, I. Krasteva, M. Marcacci, M. Ancora, A. Ciammaruconi, B. Gentile, F. Lista, A. Pini, M. Scacchia, F. Sacchini, C. Cammà

**Affiliations:** a Istituto Zooprofilattico Sperimentale dell’Abruzzo e del Molise G. Caporale, Teramo, Italy; b Army Medical and Veterinary Research Center, Rome, Italy

## Abstract

Mycoplasma mycoides subsp. mycoides is generally considered one of most pathogenic Mycoplasma species, and it is the etiological agent of contagious bovine pleuropneumonia (CBPP). Here, we present the annotated genome sequence of M. mycoides subsp. mycoides Italian strain 57/13, isolated in 1992 during CBPP outbreaks in Italy.

## GENOME ANNOUNCEMENT

Mycoplasma mycoides subsp. mycoides is the etiological agent of contagious bovine pleuropneumonia (CBPP) (1), which is considered by the World Organisation for Animal Health (Office International des Epizooties [OIE]) to be one of most severe infectious animal diseases affecting sub-Saharan-African countries, causing major losses of animals with subsequent socioeconomic repercussions, with a particular emphasis in international trades (2).

Here, we present the whole-genome sequence of the M. mycoides subsp. mycoides strain 57/13 that was isolated from the lung of a cow showing CBPP pathological lesions in 1992, during a CBPP outbreak in northern Italy. The strain was identified by PCR as M. mycoides subsp. mycoides (3). The biochemical and proteomic characterizations of the strain have been reported, but none of the Italian strains, which are considered less pathogenic than are the African strains, have been fully sequenced so far (4, 5).

The strain was grown as previously described (6). Genomic DNA was extracted from culture using the Maxwell 16 cell DNA purification kit (Promega) and sequenced by the PGM Ion Torrent platform, obtaining 427,523 single-end reads spanning from 20 to 400 nucleotides. The quality-filtered reads were assembled into contigs and annotated using a dedicated workflow available at the Orione website (7), which uses SPAdes (8) and Prokka, respectively (9). Finishing and annotation were manually curated.

The M. mycoides subsp. mycoides strain 57/13 genome contains a 1,192,498-nucleotide chromosome with 2 rRNA operons, 30 tRNAs, and 1,077 protein-coding genes, of which 332 encode hypothetical proteins conserved among other M. mycoides subsp. mycoides isolates. Compared to the M. mycoides subsp. mycoides PG1 type strain (GenBank accession no. BX293980), the M. mycoides subsp. mycoides strain 57/13 showed an overall identity of 99% and one major insertion. Genome organization highlighted a high degree of overlapped genes. As with other European strains, this isolate lacks the *gtsC* and *lppB* genes (10).

### Nucleotide sequence accession number.

The whole-genome assembly has been deposited at GenBank under accession no. CP010267.
